# The Rab6 post-Golgi secretory pathway contributes to herpes simplex virus 1 (HSV-1) egress

**DOI:** 10.1128/jvi.00599-24

**Published:** 2024-08-13

**Authors:** Melissa H. Bergeman, Kimberly Velarde, Hailee L. Hargis, Honor L. Glenn, Ian B. Hogue

**Affiliations:** 1 ASU-Banner Neurodegenerative Disease Research Center, Biodesign Institute, Arizona State University, Tempe, Arizona, USA; 2 School of Life Sciences, Arizona State University, Tempe, Arizona, USA; 3 Center for Structural Discovery, Biodesign Institute, Arizona State University, Tempe, Arizona, USA; Northwestern University Feinberg School of Medicine, Chicago, Illinois, USA

**Keywords:** herpesviruses, herpes simplex virus, fluorescent image analysis, exocytosis, egress, membrane transport, secretory pathway

## Abstract

Herpes simplex virus 1 (HSV-1) is an alpha herpesvirus that infects a majority of the world population. The mechanisms and cellular host factors involved in the intracellular transport and exocytosis of HSV-1 particles are not fully understood. To elucidate these late steps in the replication cycle, we developed a live-cell fluorescence microscopy assay of HSV-1 virion intracellular trafficking and exocytosis. This method allows us to track individual virus particles and identify the precise moment and location of particle exocytosis using a pH-sensitive reporter. We show that HSV-1 uses the host cell’s post-Golgi secretory pathway during egress. The small GTPase, Rab6, binds to nascent secretory vesicles at the *trans*-Golgi network and plays important, but non-essential, roles in vesicle traffic and exocytosis at the plasma membrane, therefore making it a useful marker of the Golgi and post-Golgi secretory pathway. We show that HSV-1 particles colocalize with Rab6a in the region of the Golgi, cotraffic with Rab6a to the cell periphery, and undergo exocytosis from Rab6a vesicles. Consistent with previous reports, we find that HSV-1 particles accumulate at preferential egress sites in infected cells. The secretory pathway mediates this preferential/polarized egress, since Rab6a vesicles accumulate near the plasma membrane similarly in uninfected cells. These data suggest that, following particle envelopment, HSV-1 egress follows a pre-existing cellular secretory pathway to exit infected cells rather than novel, virus-induced mechanisms.

**IMPORTANCE:**

Herpes simplex virus 1 (HSV-1) infects a majority of people. It establishes a life-long latent infection and occasionally reactivates, typically causing characteristic oral or genital lesions. Rarely in healthy natural hosts, but more commonly in zoonotic infections and in elderly, newborn, or immunocompromised patients, HSV-1 can cause severe herpes encephalitis. The precise cellular mechanisms used by HSV-1 remain an important area of research. In particular, the egress pathways that newly assembled virus particles use to exit from infected cells are unclear. In this study, we used fluorescence microscopy to visualize individual virus particles exiting from cells and found that HSV-1 particles use the pre-existing cellular secretory pathway.

## INTRODUCTION

During the late stages of viral replication, herpes simplex virus 1 (HSV-1) particles traffic to the plasma membrane to undergo exocytosis and complete the infectious cycle. The details of this process, and whether HSV-1 uses pre-existing cellular pathways or induces novel virus-induced pathways, are little understood. Here, we describe how HSV-1 particles use the Rab6 Golgi and post-Golgi secretory pathway to traffic from the region of the Golgi to the plasma membrane for viral egress by exocytosis.

The *trans*-Golgi network (TGN) serves as a major sorting and transportation station for cellular cargo whose final destination is the plasma membrane. After budding from the TGN, secretory vesicles traffic to the plasma membrane, where they fuse at preferential egress sites (1). There are multiple pathways that such cargo may take in this transportation network in polarized epithelial cells, fibroblasts, and neurons (2). One subset of these TGN-plasma membrane secretory vesicles is marked by the small Rab GTPase Rab6 (3).

A member of the Ras GTPase superfamily, Rab6, is a highly conserved protein across eukaryotes (4). As molecular switches, Rab proteins alternate between an active form that binds GTP and an inactive form that binds GDP. In their GTP-bound active form, Rab proteins localize to particular intracellular membranes and function to recruit a wide variety of effector proteins that determine organelle identity and direct vesicular traffic between organelles (4–6). Rab6 is a well-established marker of the Golgi apparatus and strongly localizes to the Golgi (7–9). Rab6 is considered one of the few *bona fide* Golgi Rab proteins as it is involved in intra-Golgi vesicular traffic, and vesicular traffic between the endoplasmic reticulum (ER) and Golgi, whereas other Rab proteins, like Rab8 and Rab11, participate in both Golgi and endocytic trafficking (10, 11). Importantly, Rab6 is present on nascent secretory vesicles that bud from the TGN and remains on these vesicles until they fuse at the plasma membrane for vesicle cargo exocytosis (7–9, 12).

Rab6a has been identified as an important host factor for replication and intracellular transport/egress of several types of viruses. Parvovirus capsids have been shown to associate with Rab6a secretory vesicles (13), and genetic screens have shown that HIV requires Rab6a for infection (14). The beta herpesvirus human cytomegalovirus (HCMV) requires Rab6a for viral protein trafficking to the viral assembly compartment (15, 16). Alpha herpesviruses varicella zoster virus (VZV) and pseudorabies virus (PRV) are associated with Rab6a, and PRV undergoes exocytosis from Rab6a secretory vesicles (17–20).

In HSV-1 infection, several Rab GTPases have been shown to contribute to glycoprotein trafficking. Rab1 is involved the transport of glycoproteins from the ER to the Golgi (21–23). Rab5 is associated with early endosomes and assists in trafficking viral proteins through endocytic pathways (24–26). Rab11 is associated with recycling endosomes but can also interface with endocytic and secretory trafficking (26). For Rab6a, Johns et al. (27) showed that Rab6 is important for glycoprotein trafficking to the plasma membrane, upstream of endocytic trafficking to the site of secondary envelopment. More specifically, RNA interference (RNAi) knockdown of Rab6a led to assembly and egress defects for HSV-1 and PRV (17, 27). Rab6a overexpression and a constitutively active mutant of Rab6a both promoted an increase of viral replication during PRV infection (17). However, it remains unclear the ways in which Rab6a contributes to alpha herpesvirus replication during assembly and egress and if it does so by participating in the trafficking of HSV-1 virus particles in secretory vesicles for egress and exocytosis. Thus, we chose Rab6a as a marker for the TGN and HSV-1 egress via the post-Golgi secretory pathway.

Using a range of imaging modalities for both fixed and live cells, we show that HSV-1 progeny virus particles colocalize with Rab6a, cotransport with Rab6a from the Golgi region to the cell periphery, and complete exocytosis from Rab6a secretory vesicles. In addition, we show that Rab6a preferentially accumulates at particular locations at the adherent corners, cell extensions, and cell–cell junctions, in both infected and uninfected cells. Our data suggest that HSV-1 egress uses the pre-existing Rab6 post-Golgi secretory pathway, rather than inducing novel viral-specific trafficking routes for egress.

## RESULTS

### HSV-1 capsids, HSV-1 glycoproteins, and Rab6a colocalize to the *trans*-Golgi region

**Fig 1:**
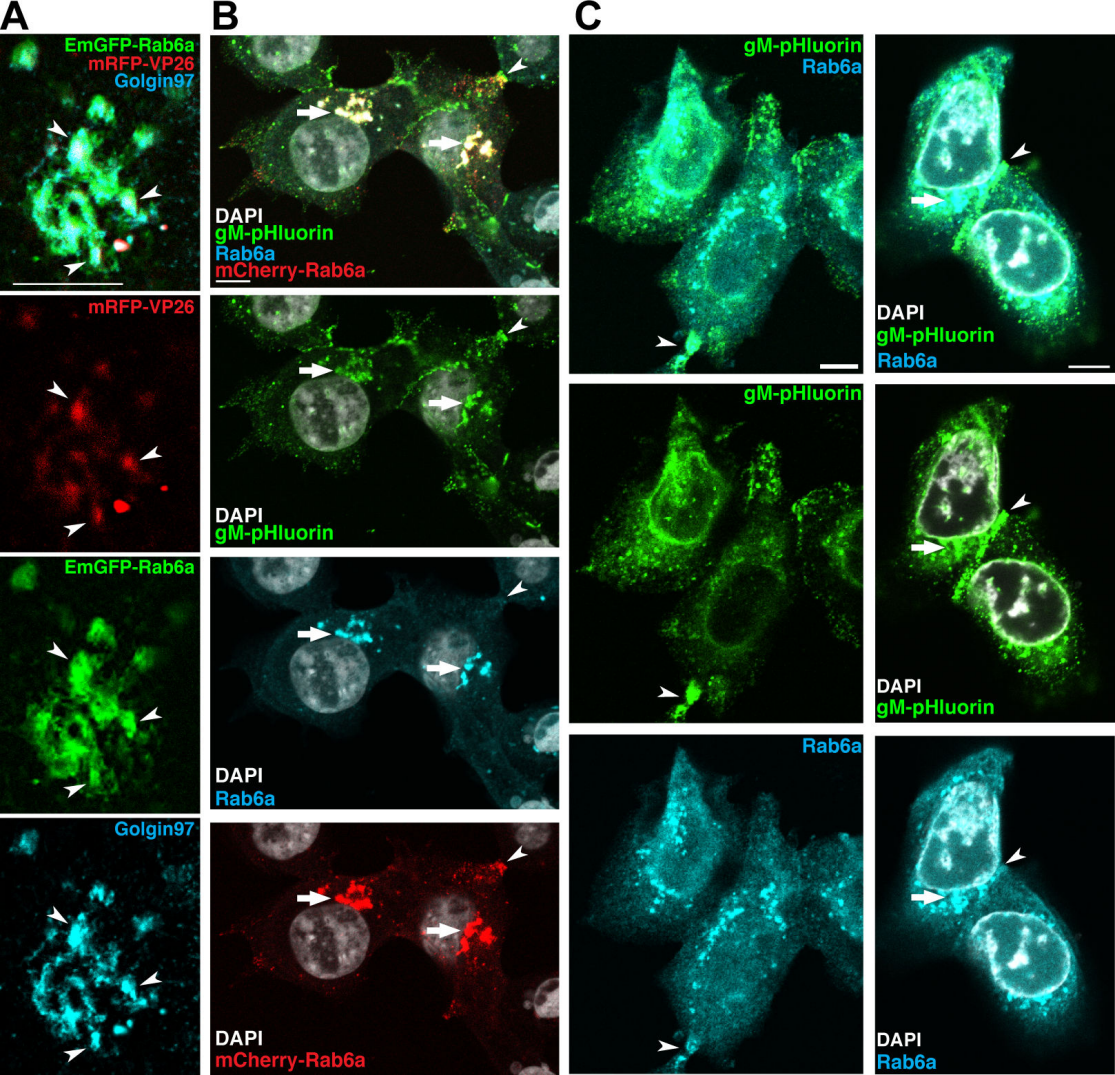
Cellular localization of Rab6a and HSV-1 proteins. (**A**) TauSTED confocal imaging shows that HSV-1 mRFP-VP26 puncta and EmGFP-Rab6a colocalize with *trans*-Golgi marker Golgin97 in Vero cells. Samples were infected with HSV-1 amplicon vector expressing EmGFP-Rab6a and HSV-1 OK14 helper virus and fixed and stained at 6 hpi. Scale bar represents 5 µm. (**B**) Confocal microscopy shows that endogenous Rab6a (blue), exogenous mCherry-Rab6a (red), and HSV-1 gM-pHluorin (green) colocalize at the Golgi (arrows) and at the cell periphery (arrowhead) in Vero cells. Cells were transduced to express mCherry-Rab6a, infected with HSV-1 IH01, and fixed and stained at 7 hpi. Scale bar represents 10 µm. (**C**) Confocal microscopy shows that endogenous Rab6a (blue) and HSV-1 gM-pHluorin protein (green) colocalize similarly in Vero cells that are not transduced and do not express exogenous Rab6a. Cells were infected with HSV-1 IH01 and fixed and stained at 7 hpi. Scale bar represents 10 µm.

We performed fluorescent TauSTED confocal microscopy on cells that had been transduced to express a fluorescent Rab6a protein and were also coinfected with a fluorescent reporter strain of HSV-1. We transduced Vero (African green monkey kidney) cells with an HSV-1-based amplicon vector that expresses an Emerald green fluorescent protein-Rab6a (EmGFP-Rab6a) transgene. The amplicon vector consists of a bacterial plasmid, transgene expression cassette, and origin of replication and packaging signal sequences from the HSV-1 genome. In the presence of a helper virus, the amplicon vector is replicated and packaged into HSV-1 particles, producing a mixed virus stock containing both amplicon and helper virus genomes. We used HSV-1 OK14 (28), based on the 17syn^+^ laboratory strain, as the helper virus because it expresses an mRFP-VP26 capsid tag to image virus particles in infected cells. Vero cells were fixed and probed for the *trans*-Golgi marker Golgin97 (29) at 6 hours post-infection (hpi). Based on our previous findings, we chose 6 hpi for this and subsequent imaging experiments because this is when the first viral progeny are produced and before the onset of cytopathic effects (30). Figure 1A represents TauSTED confocal microscopy of the juxtanuclear cluster of Golgi membranes, revealing a close association between EmGFP-Rab6a and the Golgin97. These results validate that our EmGFP-Rab6a construct does localize to and serves as a marker of *trans*-Golgi membranes (Fig. 1A).

With the findings reaffirming the localization of Rab6a to the Golgi region, we immunostained cells for endogenous Rab6a to see if exogenous Rab6a colocalized to the same cellular regions (Fig. 1B). We found that endogenous Rab6a and exogenous mCherry-Rab6a, delivered with an adenovirus vector to the Vero cells, colocalized strongly in the juxtanuclear region (arrows) (Fig. 1B). In addition, we infected the same cells with HSV-1 IH01 that expresses the pH-sensitive protein pHluorin on the first extracellular loop of envelope glycoprotein M (gM) (30). Our previous study showed that virus particles complete assembly and egress from infected cells and begin forming virus particle accumulations at preferential egress sites as early as 6 hpi (30). Though pHluorin is pH-sensitive, the process of fixing and staining the cells equilibrates intracellular pH to that of the extracellular buffer, and thus pHluorin exhibits consistent green fluorescence. At the 6 hpi timepoint, gM-pHluorin appears in the juxtanuclear region (arrows) and in the cell periphery (arrowhead). The gM-pHluorin signal is also colocalized with both the endogenous Rab6a and the exogenous Rab6a in the juxtanuclear region (Fig. 1B). These findings were also replicated in a separate set of immunostained cells. These were probed for endogenous Rab6a and infected with HSV-1 IH01. As shown in Fig. 1C, HSV-1 gM-pHluorin and endogenous Rab6a were found in the juxtanuclear region (arrow), the cell periphery and cell–cell junctions (arrowheads) in cells that were not transduced to express exogenous Rab6a.

As Rab6a functions in the post-Golgi secretory pathway to assist transporting cellular cargo from the Golgi region to the plasma membrane, it was not unexpected to find Rab6a in the juxtanuclear, Golgi region or at the cell periphery. However, the colocalization of Rab6a with HSV-1 capsid or envelope glycoproteins led us to hypothesize that HSV-1 travels to the site of exocytosis via the Rab6a pathway.

### Rab6 secretory vesicles accumulate at peripheral exocytosis sites

**Fig 2:**
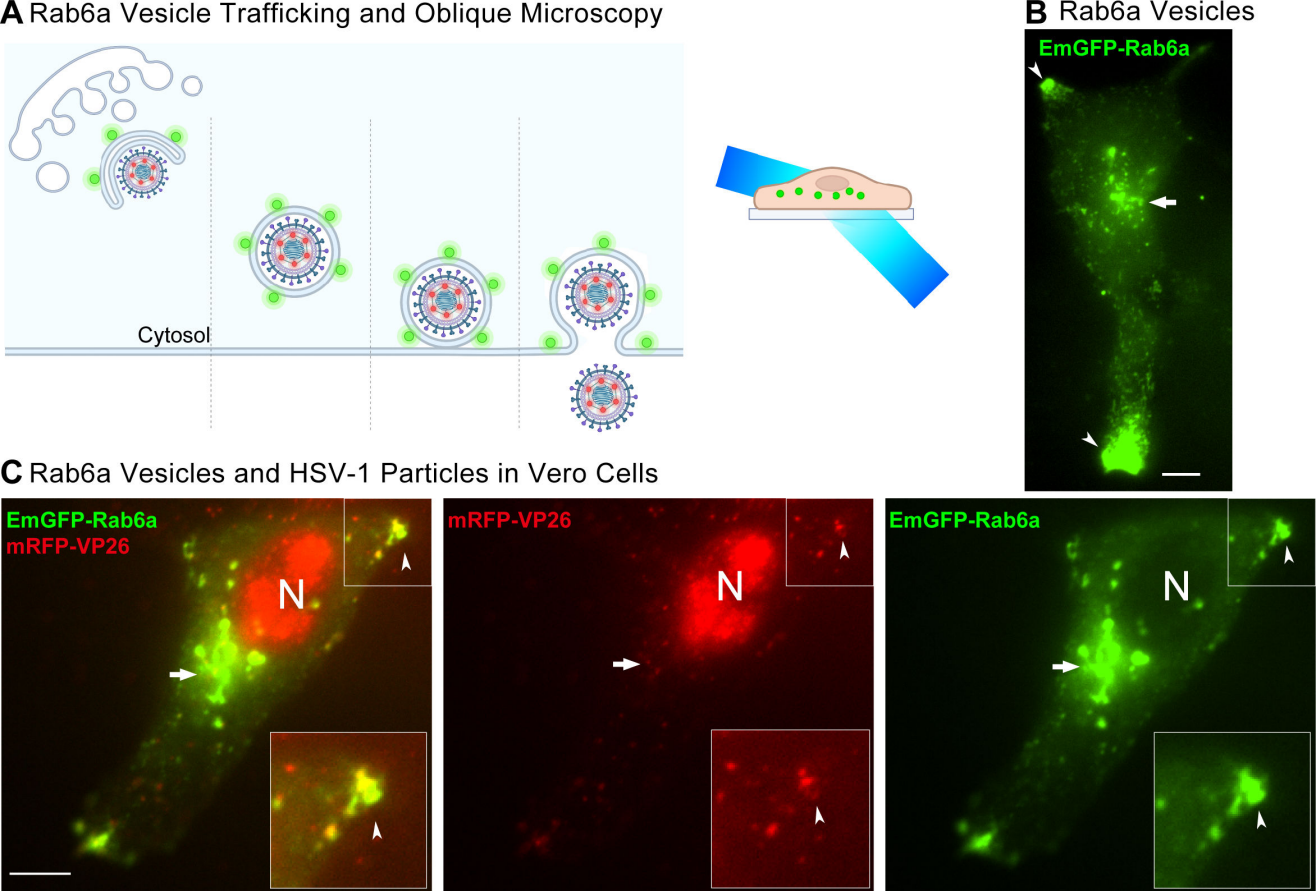
Oblique microscopy of Rab6a Golgi and secretory vesicles in uninfected and infected Vero cells. Representative images from 27 cells across eight experimental replicates. Scale bars are 10 µm. (**A**) Schematic of viral egress and fluorescent reporters. Secretory vesicles marked with EmGFP-Rab6a (green) transport virus particles marked with mRFP-VP26 (red) to the plasma membrane. Oblique microscopy projects a light sheet that excites fluorescent molecules within a thin section of the cell volume. (**B**) Cells are transduced with an HSV-1 amplicon vector expressing EmGFP-Rab6a, but are not productively infected with HSV. Rab6a localizes to the juxtanuclear Golgi region (arrow) and accumulates forming clusters at the cell periphery (arrowheads) at 6 hpi. (**C**) Cells were infected with an HSV-1 amplicon vector expressing EmGFP-Rab6a and HSV-1 OK14 helper virus and imaged at 6 hpi. EmGFP-Rab6a (green) localizes to the juxtanuclear Golgi region (arrow), and HSV-1 capsids (red) colocalize with Rab6a in clusters at the cell periphery (arrowhead), at 6 hpi (Movie S1).

Previously, we and others have shown that HSV-1 particles exit from infected cells at preferential sites (30–34). Specifically, we showed that individual HSV-1 virus particles preferentially traffic to the egress site and complete exocytosis at the plasma membrane, gradually forming large accumulations of virus particles over time (30). To assess the intracellular localization and trafficking of Rab6a secretory vesicles, we transduced Vero cells with the HSV-1 EmGFP-Rab6a amplicon vector and imaged the cells with oblique microscopy at 6 hpi. Oblique microscopy, which is sometimes called “highly inclined and laminated optical” (HiLo) light sheet microscopy or “dirty TIRF,” is similar to total internal reflection fluorescence (TIRF) microscopy. In this modality, the excitation laser is projected into a light sheet that selectively illuminates a thin section of the cell (Fig. 2A). Using this method, we were able to project the light sheet to different slices of the cell by varying the incident angle of the excitation laser. By projecting the light sheet further away from the plasma membrane with oblique microscopy, we observed that EmGFP-Rab6a strongly localizes to juxtanuclear organelles which, based on immunostaining (Fig. 1), is the Golgi and TGN (Fig. 2B, arrow). In live cells that are only positive for the EmGFP-Rab6a amplicon and do not have detectable mRFP-VP26 capsid protein expression (Fig. 2B), we also observed accumulations of Rab6a at the cell periphery (Fig. 2B, arrowheads), consistent with the results of the TauSTED and confocal microscopy that we performed (Fig. 1). Thus the accumulation of the Rab6a secretory vesicles at the cell periphery is not a result of productive HSV-1 infection (Fig. 2B).

In cells that were transduced with the EmGFP-Rab6a amplicon and infected with HSV-1 OK14, large amounts of mRFP-VP26 capsids accumulate in the nucleus, some particles colocalize with EmGFP-Rab6a in the juxtanuclear Golgi region, and particles begin to appear at sites of EmGFP-Rab6a accumulations at the cell periphery by 6 hpi (Fig. 2C; Movie S1). Importantly, virus particles and secretory vesicles appear to form accumulations in similar locations in single-positive or double-positive cells, indicating that HSV-1 particles and Rab6a secretory vesicles share similar trafficking routes and intracellular localization mechanisms (Fig. 2B and C).

### Colocalization of Rab6a secretory vesicles and HSV-1 in human fibroblast MRC5 cells

**Fig 3:**
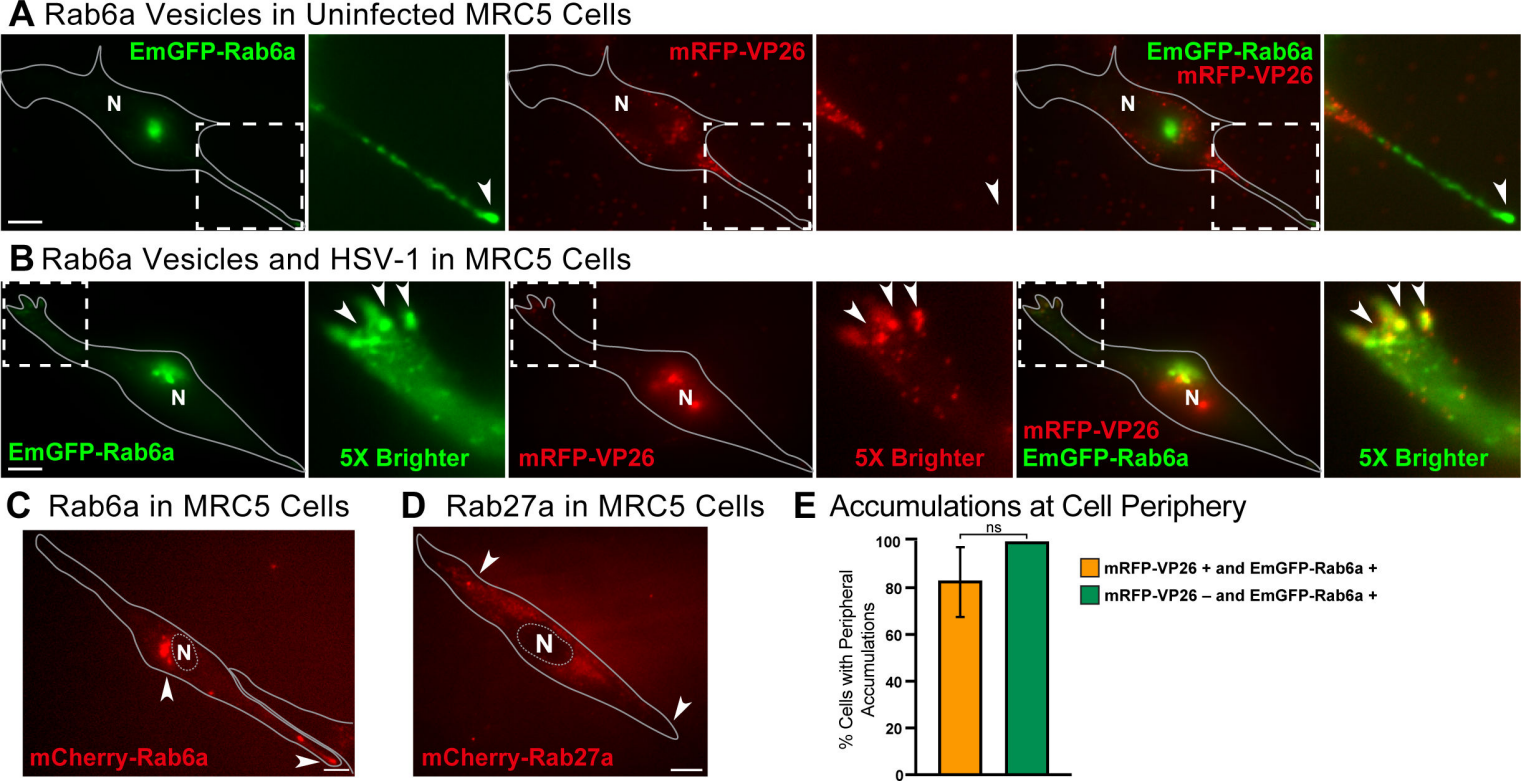
Oblique microscopy of Rab6a Golgi and secretory vesicles in uninfected and infected MRC5 human fibroblast cells. Representative images from 10 cells across four experimental replicates. Scale bars = 10 µm. (**A**) Cells are transduced with an HSV-1 amplicon vector expressing EmGFP-Rab6a (green), but are not productively infected with HSV-1, due to lack of mRFP-VP26 in the nucleus (N). Rab6a localizes to the juxtanuclear Golgi region and accumulates forming clusters at the cell periphery (arrowheads) at 6 hpi (Movie S2). (**B**) Cells were infected with an HSV-1 amplicon vector expressing EmGFP-Rab6a and HSV-1 OK14 helper virus and imaged at 6 hpi. EmGFP-Rab6a (green) localizes to the juxtanuclear Golgi region, and HSV-1 capsids (red) colocalize with Rab6a in clusters at the cell periphery (arrowhead), at 6 hpi. (**C**) Cells were transduced with an adenovirus vector to express mCherry-Rab6a. Rab6a localizes to the juxtanuclear Golgi region and accumulates forming clusters at the cell periphery (arrowheads). (**D**) Cells were transduced with an adenovirus vector to express mCherry-Rab27a. Rab27a does not localize to the juxtanuclear region or form peripheral accumulations in this cell type. (**E**) Percentage of cells with peripheral accumulations of Rab6a, comparing coinfected vs mock-infected cells. ns = no significant difference, *P* > 0.05 by Fisher’s exact test.

To investigate the role of HSV-1 egress in an additional cell type, we infected human MRC5 fibroblasts with the EmGFP-Rab6a amplicon and HSV-1 OK14 (35). At 6 hpi, we imaged cells with oblique microscopy. In MRC5 cells that were positive for only expression of EmGFP-Rab6a, whose nuclei did not have detectable mRFP-VP26 capsid protein to indicate HSV-1 infection, Rab6a accumulates at the cell periphery and in the juxtanuclear region (Fig. 3A; Movie S2). These fibroblasts exhibit an extended, elongated cell phenotype, compared to Vero cells, and the accumulation of Rab6a tends to occur at the tips of these extensions (Fig. 3A to C). In coinfected cells, we found that HSV-1 capsids colocalize in the peripheral tips with Rab6a and capsids in the nucleus indicate robust HSV-1 infection (Fig. 3B). We also transduced MRC5 cells with an adenovirus vector so that they expressed exogenous mCherry-Rab6a or mCherry-Rab27a as a control due to its role in endosomal trafficking (36). At 6 hpi, we imaged the transduced cells with TIRF microscopy. In the absence of HSV-1 infection, Rab6a accumulates in the juxtanuclear Golgi region with punctae at the cell periphery, whereas Rab27a does not (Fig. 3C and D). We quantified the number of MRC5 cells that we imaged by counting the number of cells that were positive for Rab6a accumulations and HSV-1 accumulations in the cell periphery. Eighty-three percent of cells that were transduced with EmGFP-Rab6a and coinfected with HSV-1 OK14 showed colocalized accumulations at the cell periphery. One-hundred percent of all cells that were transduced with EmGFP-Rab6a and negative for HSV-1 OK14 had accumulations of Rab6a in the cell periphery (Fig. 3E).

### HSV-1 particles and Rab6 secretory vesicles cotraffic from the Golgi region to the plasma membrane

After exiting the nucleus, HSV-1 capsids transport to the cellular membranes where secondary envelopment occurs. While the organelles that contribute to HSV-1 secondary envelopment are not entirely clear, viral membrane proteins do traffic through the TGN and colocalize with viral tegument proteins and capsids, and therefore, the TGN or TGN-derived secretory organelles are likely where secondary envelopment occurs (23, 37–40).

**Fig 4:**
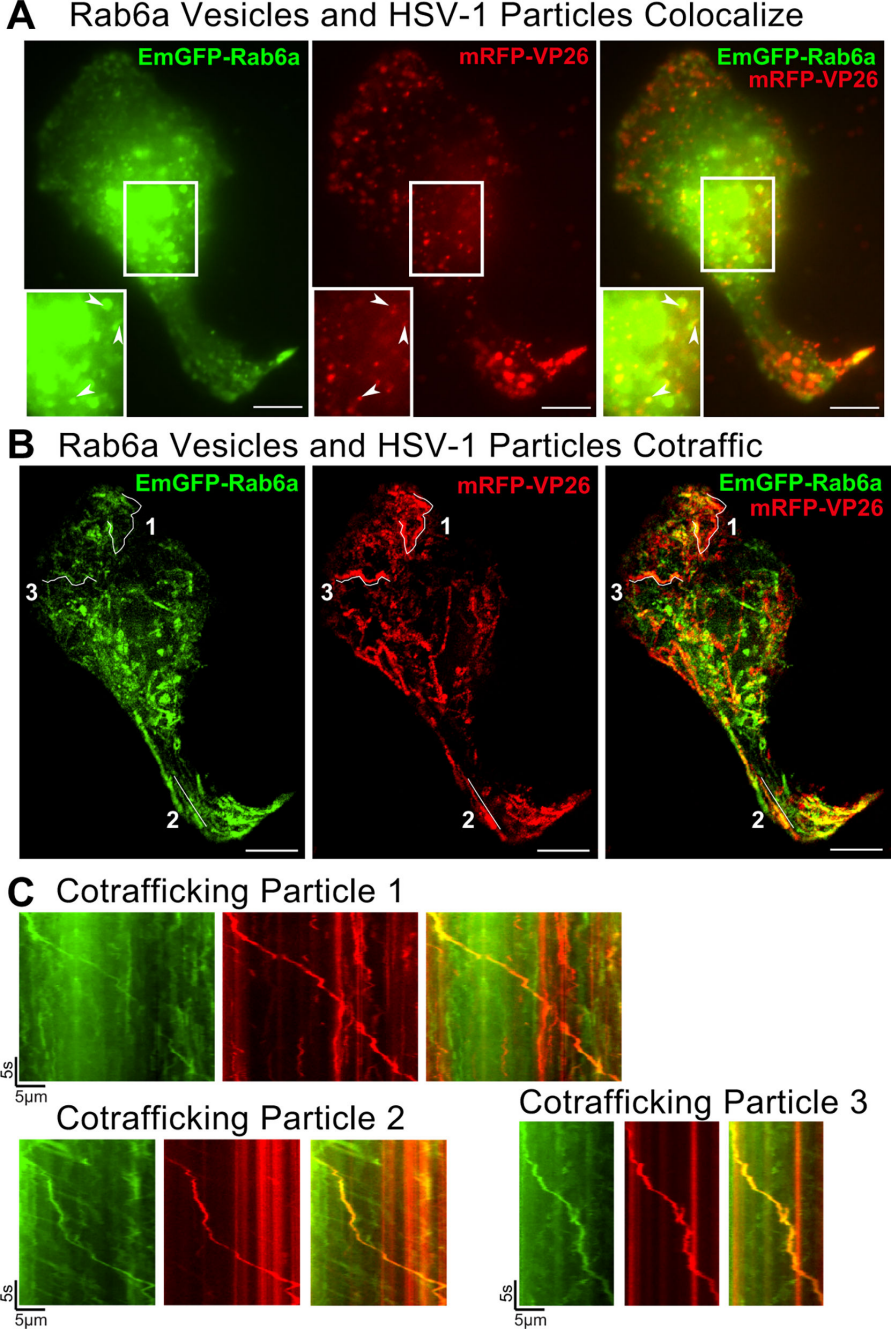
HSV-1 particles colocalize and cotraffic with Rab6a toward the cell periphery. Vero cells were infected with an HSV-1 amplicon vector expressing EmGFP-Rab6a and HSV-1 OK14 helper virus and imaged by live-cell oblique microscopy at 6 hpi. Representative images from 37 cells imaged across eight experimental replicates. Scale bars = 10 µm. (**A**) EmGFP-Rab6a (green) and mRFP-VP26 capsids (red) colocalize in distinct puncta in the Golgi region (Movie S3). (**B**) EmGFP-Rab6a (green) and mRFP-VP26 capsids cotraffic toward the cell periphery, shown as a maximum difference projection. (**C**) Kymographs of representative particle tracks indicated in panel B.

To assess whether virus particles cotraffic with Rab6 secretory vesicles, we coinfected Vero cells with HSV-1 OK14 and the EmGFP-Rab6a amplicon and imaged by live-cell TIRF microscopy so that the cytoplasm near the plasma membrane was visible, but structures deeper in the cell, such as the nucleus, were largely excluded. As above, capsids were found in the juxtanuclear Golgi region, including colocalizing with discrete EmGFP-Rab6a puncta (Fig. 4A; Movie S3). Over the course of several minutes, we observed that EmGFP-Rab6a vesicles and mRFP-VP26 capsids traffic together toward the cell periphery, as shown using maximum difference projections and kymographs (Fig. 4B and C) of the same infected cell shown in Fig. 4A. Maximum difference projections highlight areas where fluorescence increases rapidly, while suppressing background fluorescence from structures that are not moving or changing over time. Kymographs show particle movement over distance on the *X*-axis and movement over time on the *Y*-axis. Three particles, marked as 1, 2, and 3 in Fig. 4B, which are positive for both EmGFP-Rab6a and mRFP-VP26, move together on their respective paths. The kymographs in Fig. 4C show the spatial–temporal aspects of these two particles, revealing that the transport of HSV-1 particles in Rab6 secretory vesicles is a dynamic process across 210 µm from the interior of the cell to its periphery, consistent with the well-established role of microtubule-based transport of progeny virus particles.

### HSV-1 particles undergo exocytosis from Rab6 secretory vesicles

**Fig 5:**
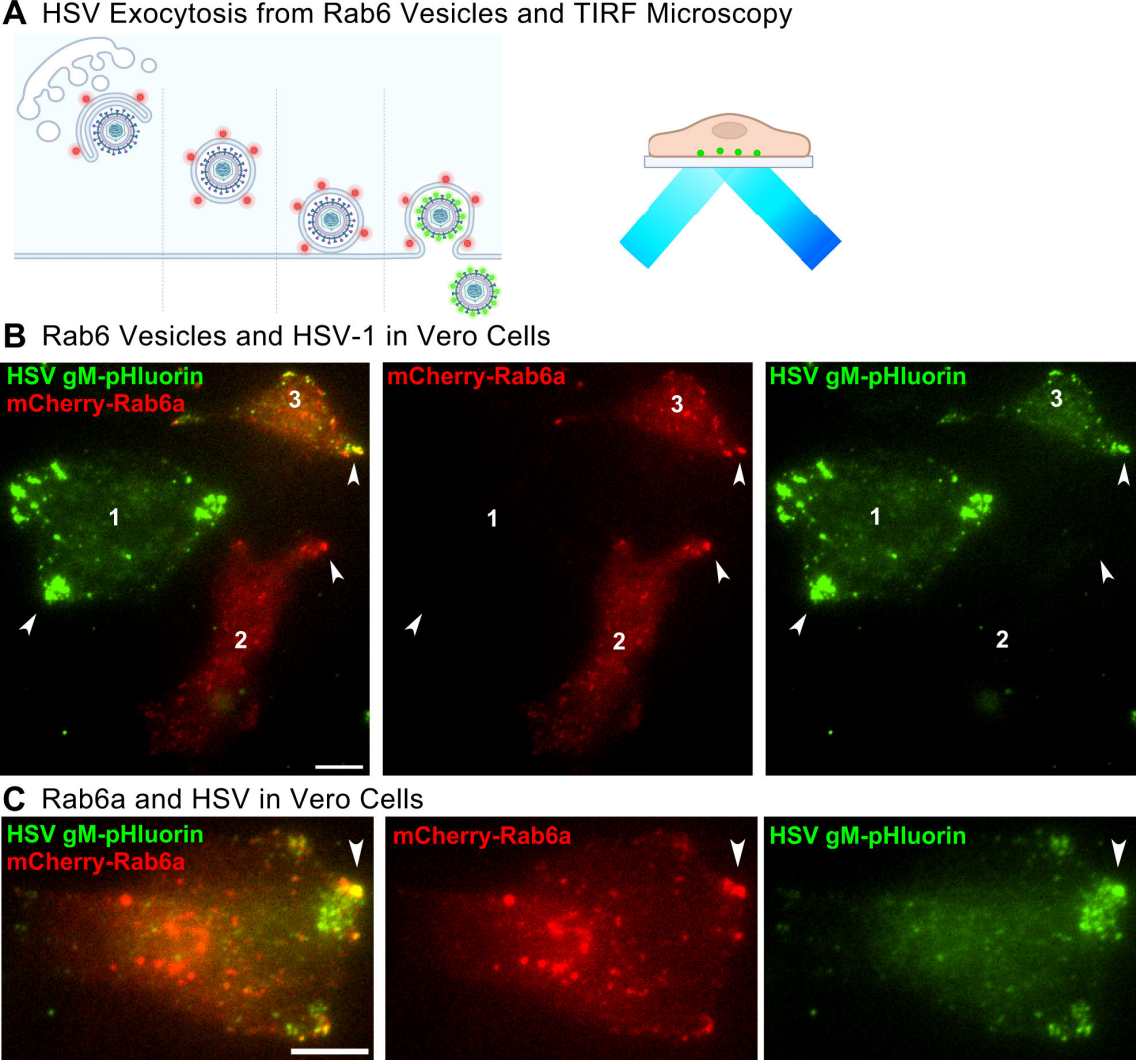
TIRF microscopy at 6 hpi of Vero cells coinfected with HSV-1 IH01 expressing gM-pHluorin (green) and an adenovirus vector expressing mCherry-Rab6a (red). Representative cell shown from 82 cells imaged across 12 experimental replicates. Scale bars = 10 µm. (**A**) Schematic of mCherry-Rab6a secretory vesicles transporting virus particles to the plasma membrane. gM-pHluorin becomes fluorescent with the pH change at the moment of exocytosis. TIRF microscopy excites fluorescent molecules at the plasma membrane (Movie S4). (**B**) HSV-1 gM accumulates in the cell periphery in cells that are not infected (1) and are infected (3). Rab6a accumulates in the cell periphery in cells that are not transduced (2) and are transduced (3). (**C**) HSV-1 particles undergo exocytosis at the cell periphery where and HSV-1 particles and Rab6a accumulate.

Since HSV-1 particles cotraffic with Rab6 post-Golgi secretory vesicles to the plasma membrane, we next wanted to determine if these intracellular trafficking events culminate in virus particle exocytosis from these vesicles. Previously, we showed that PRV particles undergo exocytosis from Rab6a vesicles in non-neuronal cells (19, 20) and from the cell body of primary neurons (18). To visualize HSV-1 exocytosis, we infected Vero cells with HSV-1 IH01, which expresses gM-pHluorin. We previously showed that gM-pHluorin is incorporated into virus particles and exhibits pH-dependent green fluorescence (30). In live cells, the lumen of secretory vesicles is acidic (pH of 5.2–5.7) (41, 42), which quenches the green fluorescence of gM-pHluorin (Fig. 5A). At the moment of exocytosis, the pHluorin moiety is exposed to the extracellular pH at the plasma membrane, and its fluorescence is dequenched (19, 20, 30, 41), allowing us to identify exocytosis events of individual light particles (L-particles) and complete virions using TIRF microscopy (Fig. 5A) (30).

Vero cells were transduced with a non-replicating adenovirus vector expressing mCherry-Rab6a, coinfected with HSV-1 IH01, and imaged by live-cell TIRF microscopy beginning at 6 hpi. HSV-1 particles containing gM-pHluorin accumulated at peripheral clusters in cells that were infected only with HSV-1 and were not positive for mCherry-Rab6a (Fig. 5B, Cell 1, Movie S4). Cells that were singly transduced, with expression of mCherry-Rab6a but no detectable HSV-1 gM-pHluorin, showed accumulations of Rab6a in peripheral clusters (Fig. 5B, Cell 2, Movie S4). In cells that were positive for both HSV-1 gM-pHluorin and mCherry-Rab6a, both accumulated in the same locations in the cell periphery (Fig. 5B, Cell 3, Movie S4; Fig. 5C).

After exocytosis, virus particles remained attached to the cell surface and largely immobile, resulting in the accumulation of large clusters of particles. Clusters of mCherry-Rab6a most likely represent secretory vesicles that have not yet fused to the plasma membrane, and clusters of dequenched, fluorescent gM-pHluorin puncta represent a combination of virions and L-particles. Because we also observed similar clustering of mRFP-VP26 capsids (Fig. 2 and 4) and clusters of colocalized gM-pHluorin and mRFP-VP26 capsids (30), a subset of these clustered particles represent complete, mature virions.

**Fig 6:**
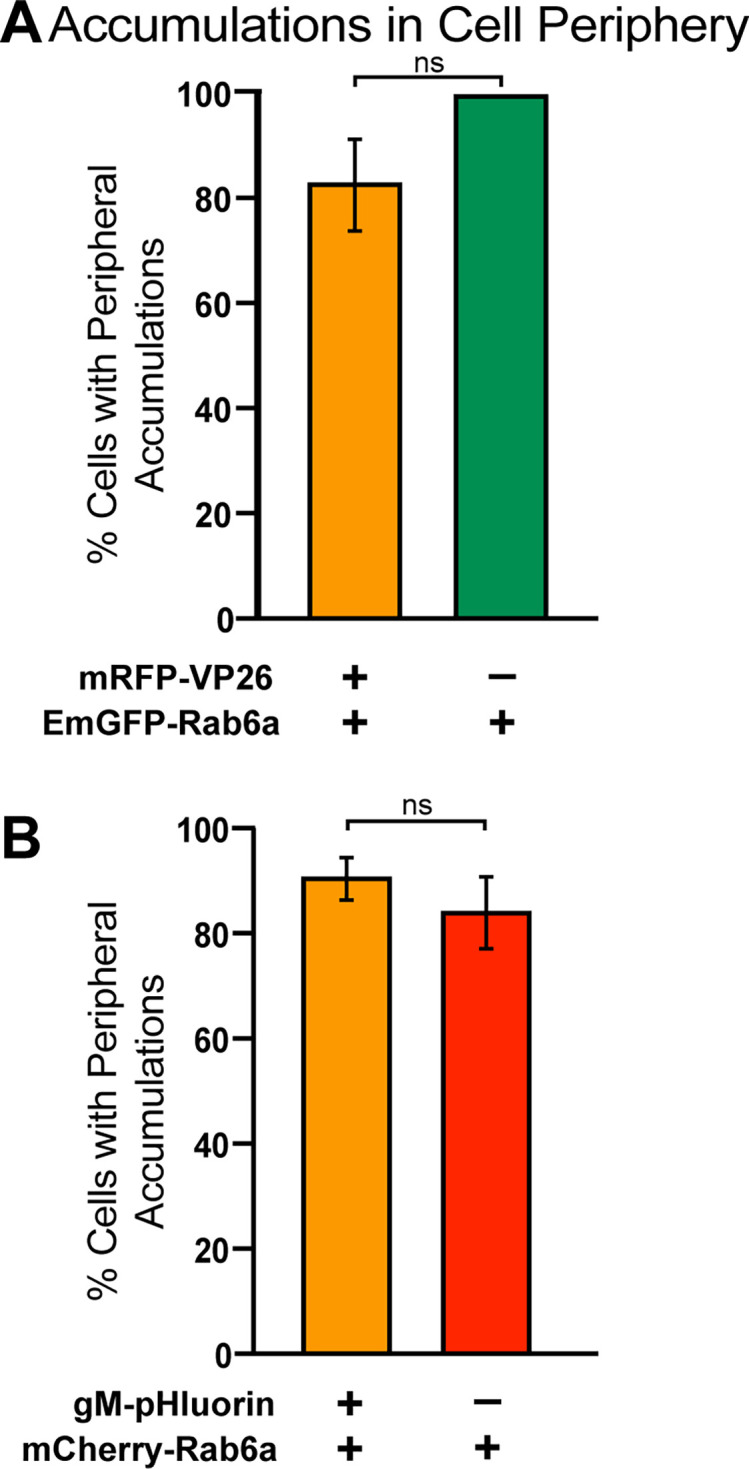
Quantification of HSV-1 and Rab6a peripheral accumulations. Percentage of cells with peripheral accumulations of Rab6a, comparing cells that are coinfected vs not productively infected with HSV-1. ns = no significant difference, *P* > 0.05 by Fisher’s exact test. (**A**) Data correspond to experiments shown in Fig. 2 and are representative of 27 cells across eight experimental replicates. (**B**) Data correspond to experiments shown in Fig. 5 and are representative of 82 cells imaged across 12 experimental replicates.

We counted all of the Vero cells imaged for Fig. 2, 4, and 5, and visually classified them based on the presence or absence of peripheral accumulations of Rab6a colocalized with viral capsid or membrane proteins. In all cases, greater than 80% of cells imaged had peripheral accumulations (Fig. 6A and B). In cells that were transduced to express EmGFP-Rab6a, 83% of cells positive for HSV-1 mRFP-VP26 capsid protein (indicating productive HSV-1 infection) and 100% of cells negative for mRFP-VP26 (indicating lack of productive HSV-1 infection) had accumulations at the cell periphery. In cells that were transduced to express mCherry-Rab6a, we found that 91% of cells positive for HSV-1 gM-pHluorin and 84% of cells negative for HSV-1 gM-pHluorin had peripheral accumulations (Fig. 6B). These data are representative of 8–12 experimental replicates in Fig. 6A and B, respectively.

### Quantification of individual exocytosis events from Rab6a vesicles

In Vero cells, identifying exocytosis of virus particles from individual Rab6a vesicles was difficult against the high background fluorescence from these large clusters of previously released particles—individual vesicles were no longer distinguishable in a large cluster (31). To overcome this problem, we transduced and infected PK15 (porcine kidney epithelial) cells, which were previously used to investigate egress of PRV (19, 20). We have shown that HSV-1 productively infects PK15 cells, but unlike in Vero cells, HSV-1 and PRV exocytosis events are uniformly distributed across the adherent cell surface, with much less accumulation of large clusters (30).

**Fig 7:**
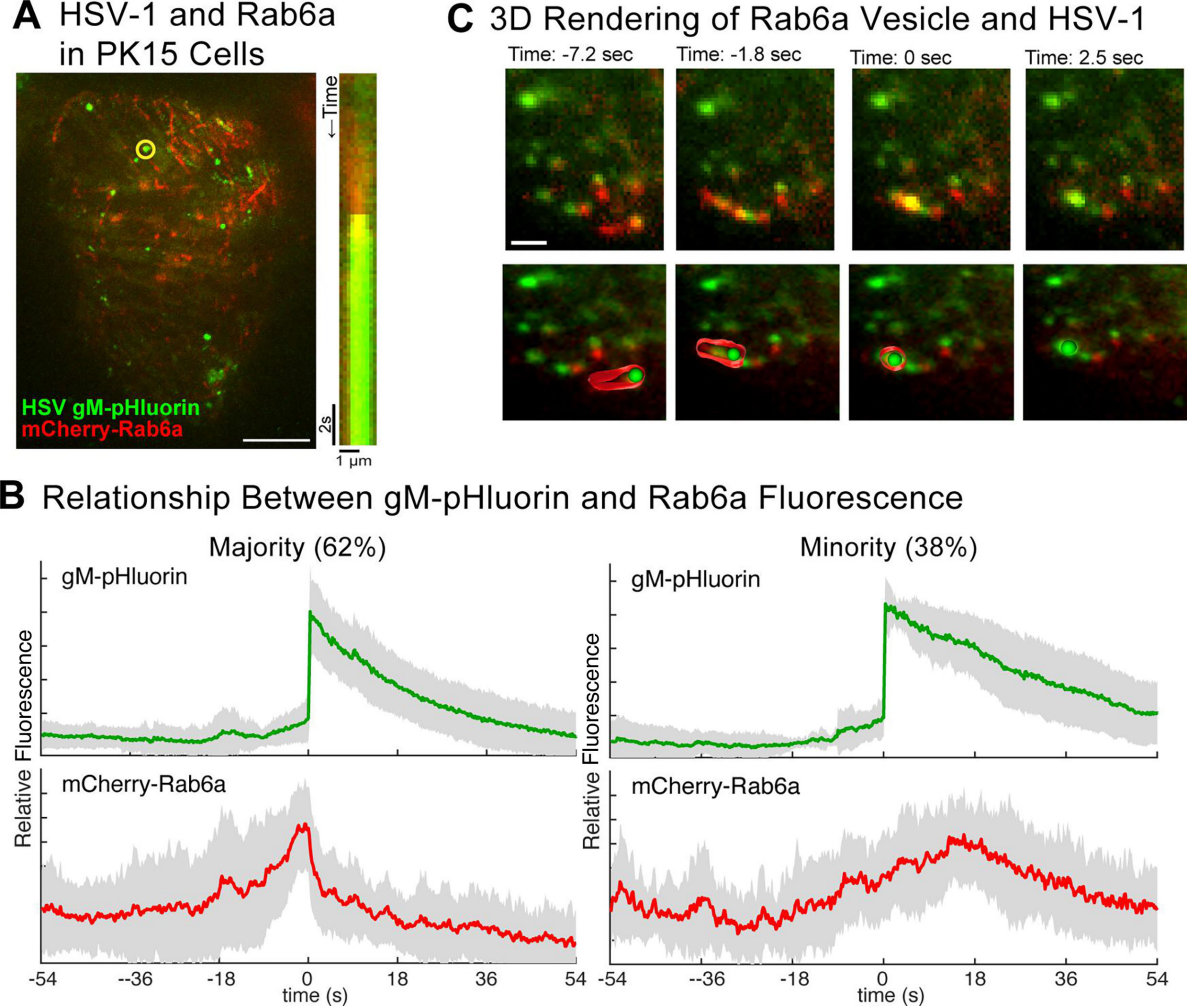
Live-cell TIRF microscopy of HSV-1 exocytosis from Rab6a vesicles. Vero cells were transduced with an adenovirus vector to express mCherry-Rab6a, infected with HSV-1 IH01 expressing gM-pHluorin and imaged beginning at 6 hpi. Images are representative of six experimental replicates. (**A**) Maximum difference projection of coinfected cell over 3:38 sec of imaging. Kymograph of particle (yellow circle) movement and fluorescence over time showing HSV-1 exocytosis from a Rab6a vesicle. Scale bar = 10 µm. (**B**) Ensemble average of gM-pHluorin (green) and mCherry-Rab6a (red) fluorescence over 45 exocytosis events. Shading represents standard deviation. (**C**) 3D rendering of a Rab6a vesicle transporting an HSV-1 particle. Top row shows still frame images at the indicated time points before, at, and after the exocytosis event (Movie S5). Bottom row shows an interpretive 3D model, tilted at ~36° on the *X*-axis, of the Rab6a vesicle and virus particle for the same time points. Scale bar = 2 µm.

Over the course of several minutes, we observed individual virus particles undergoing exocytosis from mCherry-Rab6a vesicles, which were easily distinguishable without the formation of large clusters. A representative exocytosis event is shown using maximum difference projection and a kymograph (Fig. 7A). Here, maximum difference projections highlight areas where fluorescence increases rapidly due to gM-pHluorin dequenching, and kymographs show the change in particle fluorescence over time on the *Y*-axis.

To quantify gM-pHluorin and mCherry-Rab6a fluorescence over many individual exocytosis events, we calculated the average relative fluorescence intensity over time. Individual exocytosis events were measured, and time series data were aligned to a common time = 0 based on the peak of gM-pHluorin fluorescence intensity. Average green fluorescence exhibits a sharp increase, representing rapid dequenching of gM-pHluorin upon exocytosis, followed by a slow exponential decay due to photobleaching. For a majority of exocytosis events (*n* = 28/44, 62%), red fluorescence gradually increased prior to exocytosis, representing the arrival of a mCherry-Rab6a-positive vesicle to the site of exocytosis. Immediately after exocytosis, the red signal rapidly decays, representing a combination of mCherry-Rab6a molecules diffusing away from the site of exocytosis and photobleaching (Fig. 7B and C; Movie S5).

However, some exocytosis events (*n* = 17/45, 38%) did not appear to be associated with a strong mCherry-Rab6a signal (Fig. 7B, Supplemental 1). The reduction or lack of mCherry-Rab6a signal before exocytosis may be a result of low mCherry-Rab6a expression and high fluorescence background, or a minority of HSV-1 particles may use alternative secretory mechanisms. While Rab6a is a marker for the TGN and post-Golgi secretory vesicles, it is possible that Rab6a does not mark all of them and that viral exocytosis also takes place from secretory vesicles that do not have Rab6a.

### Secretory Rab27a and endocytic Rab5a are not associated with HSV-1 exocytosis

As negative controls, we also tested Rab27a and Rab5a. Many enveloped viruses, including HSV-1 (43), use cellular ESCRT machinery for virion budding or membrane scission. In uninfected cells, ESCRT produces intralumenal vesicles in late endosomes/multivesicular bodies. Rab27a regulates the exocytosis of endosomal organelles, including multivesicular bodies, to produce extracellular exosomes. Rab27 has been implicated in HSV-1 secondary envelopment, specifically in an oligodendrocyte cell line where Rab27 appears to colocalize with the trans-Golgi (36). Rab5 is present on early endosomes and has also been implicated in HSV-1 secondary envelopment (26, 27).

**Fig 8:**
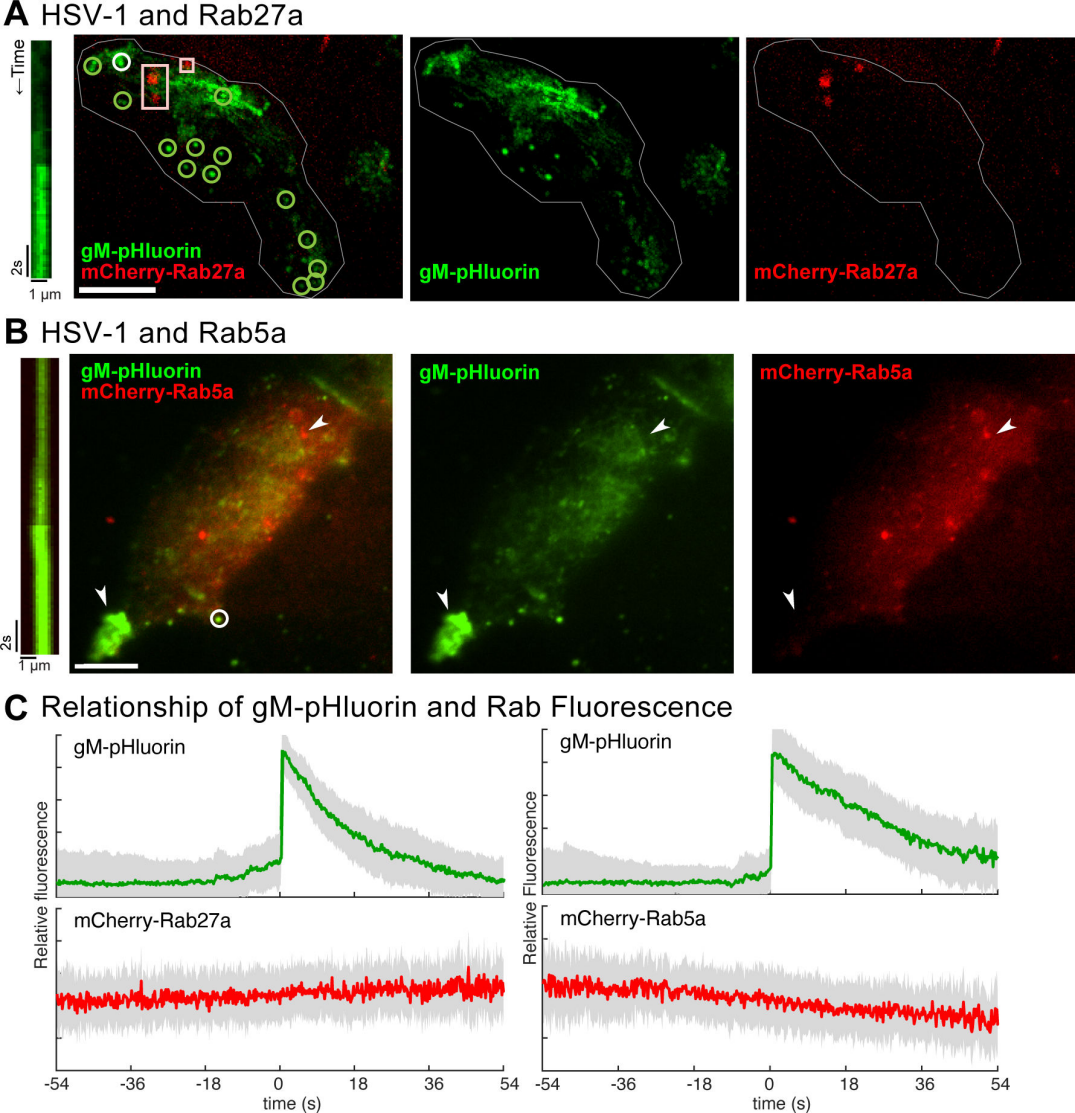
Live-cell TIRF microscopy of HSV-1 exocytosis, which is not associated with Rab5a or Rab27a. Vero cells were transduced with an adenovirus vector expressing mCherry-tagged Rab proteins (red), infected with HSV-1 IH01 expressing gM-pHluorin, and imaged beginning at 6 hpi. Scale bars = 10 µm. (**A**) Representative cell expressing mCherry-Rab27a and infected with HSV-1 IH01. Individual virus particle exocytosis events are marked with green circles, and Rab27a vesicles are marked with red boxes. The viral exocytosis event illustrated in the kymograph is marked with a white circle. Representative images from 10 cells imaged across four experimental replicates. (**B**) Representative cell expressing mCherry-Rab5a and infected with HSV-1 IH01 (Movie S6). A peripheral accumulation of dequenched gM-pHluorin and a Rab5a vesicle are indicated (arrowheads). The viral exocytosis event illustrated in the kymograph is marked with a white circle. Representative images from 13 cells imaged across two experimental replicates. (**C**) Ensemble average of gM-pHluorin (green) and mCherry-Rab (red) fluorescence over time. Shading represents standard deviation. Data are representative of 55 exocytosis events (mCherry-Rab27a) or 63 exocytosis events (mCherry-Rab5a).

To investigate the role of these Rab GTPases, we transduced Vero cells with adenovirus vectors expressing mCherry-Rab27a or mCherry-Rab5a, then infected with HSV-1 expressing gM-pHluorin, and imaged by TIRF microscopy beginning at 6 hpi. As shown in Fig. 8A, mCherry-Rab27a localizes to discrete clusters (boxes), and HSV-1 particles undergo exocytosis from these transduced cells (circles). However, mCherry-Rab27a does not colocalize with gM-pHluorin (Fig. 8A) and does not appear to be associated with viral exocytosis (Fig. 8C). Similarly, mCherry-Rab5a localizes to distinct puncta scattered throughout the cell, but does not appear to strongly colocalize with gM-pHluorin (Fig. 8B; Movie S6) and does not appear to be associated with viral exocytosis (Fig. 8C). These results show that colocalization with Rab GTPases is specific to stages of viral replication and not merely an artifact of transduction or HSV-1 infection. While Rab27 may play important roles in specialized cell types and Rab5 may be important for viral glycoprotein trafficking upstream of HSV-1 egress, these Rabs do not appear to be associated with virus particle exocytosis.

### Effect of Rab6a inhibition on HSV-1 replication

Several studies have now shown that Rab6a is involved in alpha herpesvirus replication and egress (17, 18, 20, 27, 40). At least two have shown that RNAi knockdown of Rab6a significantly reduces virus production but with effect sizes ranging from a modest 0.5 log reduction to ~2 log reduction in virus titer (17, 27). To determine whether Rab6 plays an essential functional role in HSV-1 egress in our microscopy assays, we transduced Vero cells with adenovirus vectors expressing mCherry-Rab6a or a GDP-locked, non-functional mCherry-Rab6a(T27N) mutant (44).

Since Rab6a serves as a Golgi and post-Golgi secretory vesicle marker, we first compared the spatial distribution of Rab6a to Rab6a(T27N) by widefield fluorescence microscopy, during HSV-1 infection at 7 hpi. Consistent with our prior figures, we found that mCherry-Rab6a strongly localizes to a juxtanuclear cluster, with some puncta and clusters at the cell periphery. In contrast, the mutant mCherry-Rab6a(T27N) was diffuse in the cytoplasm, consistent with its loss of function mutation. To assess the localization of HSV-1, we categorized 50 infected cells from each experimental condition in two ways, based on the intracellular localization of gM-pHluorin: first, if gM-pHluorin localized most strongly to a juxtanuclear cluster (similarly to Fig. 1C or Fig. 2C), we inferred that gM-pHluorin was accumulating at Golgi membranes. In contrast, if gM-pHluorin localized most strongly in a nuclear envelope and reticular pattern (similarly to Fig. 1B), we inferred that gM-pHluorin was retained at the ER. Second, we also classified cells based on the formation of peripheral clusters of gM-pHluorin at cell extensions and cell–cell junctions (as shown in Fig. 1B, 2B and C).

**Fig 9:**
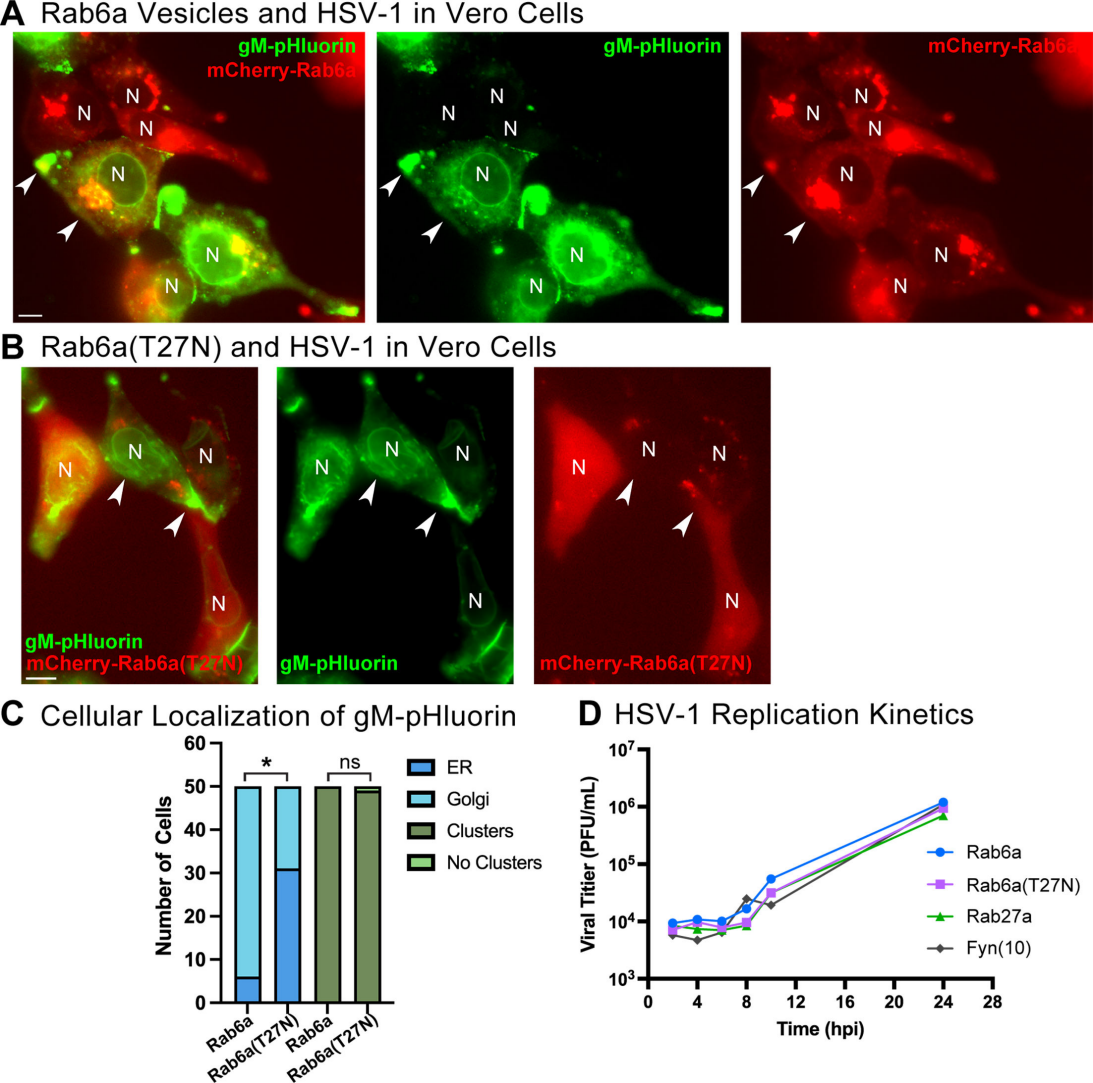
A non-functional GDP-locked Rab6a mutant affects gM-pHluorin localization, but does not affect HSV-1 replication. (**A–C**) Vero cells were transduced with adenovirus vectors expressing mCherry-Rab6a or (T27N) mutant, infected with HSV-1 IH01, and imaged by widefield fluorescence microscopy at 7 hpi. gM-pHluorin localizes to ER membranes, visible as a distinct ring around the nucleus (N), juxtanuclear Golgi, and accumulates forming clusters at the cell periphery (arrowheads). Scale bars = 10 µm. (**A**) mCherry-Rab6a localizes to the juxtanuclear Golgi region and peripheral accumulations. (**B**) GDP-locked mCherry-Rab6a(T27N) does not form discrete puncta and is diffuse throughout the cell. (**C**) Cells (*n* = 50) were scored based on whether gM-pHluorin localizes predominantly to ER or the juxtanuclear Golgi region and whether gM-pHluorin accumulates to form peripheral clusters. Expression of Rab6a(T27N) caused a significant increase (*P* < 0.001) in ER localization but no significant difference in peripheral accumulations (ns; *P* > 0.05) by Fisher’s exact test. (**D**) Single-step replication kinetics of HSV-1 in Vero cells transduced with adenovirus vectors expressing mCherry-Rab6a, mCherry-Rab6a(T27N), mCherry-Rab27a, or mCherry-Fyn(10). Data represent geometric means of three experimental replicates.

With expression of the mutant mCherry-Rab6a(T27N), we found that there was statistically significant greater localization of gM-pHluorin to the ER, consistent with a defect in Golgi function (Fig. 9A to C). Nevertheless, in nearly all infected cells, we still observed peripheral accumulations of gM-pHluorin at cell protrusions and cell–cell junctions (Fig. 9C), raising the possibility that secondary envelopment, trafficking, and egress of HSV-1 may not be strongly impacted by the Rab6a GDP-locked mutant.

To determine whether the Rab6a mutant affects HSV-1 replication and egress, we also performed a single-step growth curve, measuring cell-free virus titer released into the supernatant. We compared mCherry-Rab6a to mCherry-Rab6a(T27N). As additional controls for the effects of adenovirus transduction or protein overexpression in general, we also transduced cells with adenovirus vectors expressing Fyn(10)-mCherry, which is myristoylated and palmitoylated for localization to the cytosolic face of cellular membranes, and mCherry-Rab27a, which was not associated with HSV-1 egress in Fig. 8. Overall, we observed little or no effect of any treatment condition (Fig. 9D). Thus, while our data indicate that HSV-1 particles use the post-Golgi secretory pathway marked by Rab6, the molecular functions of Rab6 *per se* may not be necessary or might be provided by redundant functions of other Rab proteins, as discussed below.

## DISCUSSION

Viral egress is a highly involved process: after assembly, virus particles must be sorted and delivered to the appropriate site for release from the infected cell by exocytosis. It has been shown via RNAi knockdown studies that Rab6a is important for HSV-1 replication. We and others have also previously shown that Rab6a secretory vesicles are involved in egress of the related alpha herpesvirus, PRV (17–20, 27, 40). By using live-cell fluorescence microscopy, we found that Rab6a is associated with the final stages of HSV-1 replication. Rab6a and HSV-1 progeny particles colocalize in the juxtanuclear region of the Golgi (Fig. 1 to 3), where they cotraffic to the plasma membrane (Fig. 4). At the plasma membrane, virus particles undergo exocytosis from Rab6a secretory vesicles (Fig. 5 to 7).

In addition to HSV-1 egress from Rab6 secretory vesicles, we also observed that HSV-1 particles and Rab6a positive secretory vesicles accumulated in clusters together at the cell periphery. This clustering of HSV-1 has been previously reported by others (31–33), including as far back as some of the earliest indirect immunofluorescence studies with HSV-1 (34). We previously described how these accumulations are preferential egress sites for HSV-1, forming over time as individual virus particles arrive at the plasma membrane and complete exocytosis from the cell (30). Preferential egress sites also tend to form at cell–cell junctions and facilitate cell–cell spread (30). Importantly, these preferential sites appear to be the result of preferential microtubule-based intracellular transport, as an exogenous kinesin microtubule motor, which accumulates near microtubule plus-ends, coaccumulates in the same locations, in infected and uninfected cells (30). In the cell biological literature, Rab6a coordinates the delivery of secretory cargoes at the plasma membrane, particularly near focal adhesions (9).

Following exocytosis, Rab GTPases diffuse in the plane of the plasma membrane, deactivate by GTP hydrolysis, and detach from the membrane, so Rab6 would not be expected to accumulate at the cell periphery after exocytosis. It is possible that this accumulation of Rab6 is an artifact of Rab6 overexpression and/or high expression of viral secretory cargoes (viral proteins and particles) during infection. This may indicate that there is a local rate-limiting amount of some other cellular factors that are necessary for vesicle fusion [e.g., soluble N-ethylmaleimide-sensitive factor attachment receptor (SNARE) proteins], deactivation and membrane dissociation of the Rab proteins (e.g., Rab GTPase-activating proteins and Rab GDP dissociation inhibitors), or endocytic processes. This coaccumulation/clustering effect may provide a facile method to identify additional cellular factors that might contribute to Rab6 and HSV-1 trafficking and egress routes and will be the subject of future studies.

However, our data also suggest that Rab6a may not be essential for HSV-1 replication: overexpressing a non-functional GDP-locked Rab6a mutant altered intracellular localization of gM-pHluorin, but did not inhibit accumulation of particles at cell protrusions and did not inhibit a single-step replication (Fig. 9). These results should not be entirely surprising: in the alpha herpesvirus literature, studies using RNAi knockdown of Rab6 saw variable effect sizes, ranging from a modest 0.5 log to a 2 log reduction in virus titer (17, 27). In a very recent study, Liang et al. (17) showed that expressing the GDP-locked Rab6a(T27N) mutant had no significant effect on replication and egress of PRV. With our HSV-1 experiments, we concur (Fig. 9).

There are several possible explanations for these findings. First, Rab6 is not essential for secretion of cellular cargoes in the cell biological literature—disruption of Rab6 can cause secretion to be somewhat reduced or delayed, or the subcellular location of exocytosis is altered, but secretion of cellular cargoes is not eliminated (9). Second, the Rab6a(T27N) mutant protein used here and in Liang et. al (17) is expressed in the presence of wild-type endogenous Rab6. Although the Rab6a(T27N) mutation is widely described as a “dominant-negative” mutation and we do see a dominant effect on the intracellular localization of gM-pHluorin (Fig. 9), this may be more of a loss-of-function mutation, rather than a true dominant-negative. Any remaining functionality of endogenous Rab6 may be sufficient for HSV-1 and PRV replication, assembly, and egress. Third, Rab6a is likely not the sole Rab GTPase involved in HSV-1 egress, and these other Rab proteins may compensate for lack of Rab6 function. Our prior studies have shown that PRV particles undergo exocytosis from secretory vesicles that are also marked by Rab8a and Rab11a (19, 20). Studies in the cell biological literature suggest that Rab proteins function combinatorially and multiple different Rabs can form a protein interaction network to redundantly recruit effector proteins. For example, Rab6, Rab8, and Rab10 may form a protein interaction network that cross-recruit many of the same interacting and effector proteins (45). Such redundancy may explain why disrupting Rab6 has limited effects on both cell biology and alpha herpesvirus assembly and egress.

Finally, there may be other secretory pathways that work in parallel. The involvement of the Rab6a secretory pathway in HSV-1 egress and exocytosis neither excludes nor precludes other assembly and egress pathways in HSV-1 replication (26, 27, 46). For example, recent work has suggested that two parallel pathways may be responsible for VZV egress: enveloped VZV particles cofractionated with Rab6, suggesting that VZV may use the Rab6 post-Golgi secretory pathway like HSV-1 and PRV, but VZV was also associated with a mannose-6-phosphate receptor trafficking pathway. We note that while a majority of HSV-1 exocytosis events were clearly associated with Rab6a, we also observed HSV-1 exocytosis events that did not have detectable Rab6 (Fig. 7). Thus, we conclude that HSV-1 uses the Rab6 post-Golgi secretory pathway for egress, but further studies will be needed to address the functional redundancy in these post-Golgi secretory mechanisms and other trafficking routes that may be used in parallel.

## MATERIALS AND METHODS

### Cell lines and cell culture

Vero (ATCC, CCL-81), human lung fibroblast MRC5 (ATCC, CCL-171), and PK15 (ATCC, CCL-33) cells were obtained from ATCC and maintained in Dulbecco's modified Eagle medium (Cytiva) supplemented with 10% fetal bovine serum (Omega and Peak Scientific) and 1% Penicillin-Streptomycin (Gibco), in a 5% CO_2_ incubator at 37°C. Human embryonic kidney (HEK) 293A cells were a kind gift from Masmudur Rahman (Arizona State University).

### HSV-1 viruses

HSV-1 IH01 was constructed and propagated as previously described (30). HSV-1 OK14 was obtained from Lynn Enquist (Princeton University) (28) from whole-genome sequenced archival stocks.

### HSV-1 amplicon vector construction and propagation

The amplicon vector plasmid pCPD-HSV-N-EmGFP-DEST was constructed by the DNASU Plasmid Repository (Biodesign Institute, Arizona State University), as follows: the plasmid HSV-DYN-hM4Di was a gift from John Neumaier (Addgene plasmid #53327) (47). Unnecessary promoter and transgene sequences were removed by digestion with HindIII and religating, to produce pCPD-HSV. This plasmid contains a HSV-1 packaging signal and Oris origin of replication. The plasmid pcDNA6.2/N-EmGFP-DEST was obtained from Invitrogen (ThermoFisher). The CMV promoter, EmGFP coding region, Gateway recombination cassette (attR1, CmR selection marker; ccdB counterselection marker, attR2), and HSV-1 TK polyadenylation signal were PCR amplified and ligated into the HindIII site on pCPD-HSV, to produce pCPD-HSV-N-EmGFP-DEST. The human Rab6a coding sequence (DNASU Plasmid Repository, #HsCD00296778, National Center for Biotechnology Information, Nucleotide reference BC096818) was inserted by Gateway recombination (Invitrogen) to make an in-frame EmGFP-Rab6a fusion.

To propagate the EmGFP-Rab6a amplicon vector, 3.5 × 10^5^ HEK 293A cells were seeded into each well of a 6-well plate (CELLTREAT), incubated overnight, and then transfected with 3 µg of amplicon plasmid using Lipofectamine 2000 (Invitrogen). Twenty-four hours after transfection, cells were infected with 10^5^ infectious units of HSV-1 OK14. Cells were incubated for another 24 hours, and then cells and supernatants were harvested. The amplicon stock was passaged at high multiplicity of infection three times on Vero cells, and cells and supernatants were harvested, and stored at −80°C.

### Adenovirus vectors

Non-replicating E1/E3-deleted adenovirus vectors expressing mCherry-Rab6a, mCherry-Rab5a, and mCherry-Rab27a were previously described (19, 20) and propagated on HEK 293A cells. The adenovirus vector expressing mCherry-Rab6a(T27N) was constructed by PCR mutagenesis and Gateway recombination and verified by Sanger sequencing (Supplemental 2). The adenovirus vector expressing Fyn(10)-mCherry (the first 10 residues of Fyn kinase, an acylation signal, fused to mCherry) was constructed by PCR amplification from previously described plasmids and Gateway recombination (48).

### Live-cell oblique and TIRF microscopy

Cultures were prepared for TIRF and oblique microscopy by seeding cells at a low density on glass-bottom 35-mm dishes (CELLTREAT, Ibidi, or MatTek), and incubated overnight before viral transduction and infection. Cells were infected with HSV-1, amplicon vectors, and/or adenovirus vectors and imaged beginning at 6 hpi. The HSV-1, amplicon vector, and adenovirus vector inoculum amounts were determined empirically to synchronously infect/transduce a majority of cells as observed by fluorescence microscopy.

Fluorescence microscopy was performed using a Nikon Eclipse Ti2-E inverted microscope in the Biodesign Imaging Core facility (Arizona State University, Tempe, AZ, USA). This microscope is equipped with TIRF and widefield illuminators, a Photometrics Prime 95B sCMOS camera, a 60× high-numerical aperture TIRF objective, and objective and stage warmers for 37^°^C live-cell microscopy. Lasers that are 488 nm and 561 nm were used to excite green and red fluorescent proteins, respectively.

### Immunofluorescence and confocal microscopy

Vero cells were seeded into 8-well Ibidi dishes and cultured overnight. Cells were infected with HSV-1 IH01, mCherry-Rab6a adenovirus vector, or cotransduced/infected, as described above. At 7 hpi, cells were fixed for 10 minutes with 4% freshly prepared paraformaldehyde in phosphate-buffered saline (PBS), permeabilized with 0.5% Triton X-100 for 90 seconds, and then fixed again for 2 minutes. After fixation, samples were rinsed three times with PBS and then blocked with 5% goat serum in PBS for 30 minutes at room temperature. Primary rabbit polyclonal antibody against Rab6a (Abcam ab95954) was used at a 1:200 dilution in antibody diluent (0.05% Tween 20, 1% goat serum, in PBS). Cells were incubated in primary antibody overnight in the dark at 4°C and then rinsed three times in wash buffer (0.05% Tween 20, in PBS). Secondary antibody, anti-rabbit AlexaFluor 633 (ThermoFisher A21071), was diluted 1:1,000 in antibody diluent and incubated on samples for 2 hours at room temperature with gentle rocking. Cells were rinsed three times with wash buffer and once with PBS. Nuclei were labeled with 0.1 µg/mL 4′,6-diamidino-2-phenylindole (DAPI) in PBS. Samples were imaged on a Nikon AX R laser scanning confocal microscope in the Biodesign Imaging Core facility (Arizona State University, Tempe, AZ, USA) using a 60 × 1.42 NA objective. The DAPI channel was excited at 405 nm, pHluorin at 488 nm, mCherry at 568 nm, and AlexaFluor 633 at 640 nm. Emissions for these channels were collected in the blue, green, red, and far-red spectral ranges, respectively.

For TauSTED microscopy, Vero cells were seeded into #1.5 glass slides, cultured overnight, and then infected with the EmGFP-Rab6a amplicon and HSV OK14. Cells were then fixed and permeabilized as desceribed above. Primary rabbit antibody against Golgin97 (Abcam) was used in a 1:250 antibody diluent. Cells were incubated overnight and rinsed three times with wash buffer. Secondary anti-rabbit Atto 647N antibody (ATTO-TEC) was diluted 1:500 in antibody diluent and incubated for 1 hour. Samples were washed three times with wash buffer, allowed to dry for 24 hours, and mounted with ProLong Diamond (Thermo Fisher Scientific). TauSTED imaging was performed on a Leica DMi8 Inverted microscope base equipped with the Leica TauSTED system at the University of California San Diego School of Medicine Microscopy Core. EmGFP was excited at 493 nm, mRFP at 568, and Atto 647N at 658 nm. Emissions were collected in the green, red, and far-red ranges, respectively.

### Image analysis

Image analysis to identify exocytosis events and HSV colocalization with Rab6a vesicles was conducted in Fiji software (49). Fluorescence microscopy images were prepared for publication using Adjust Brightness/Contrast, Reslice (to produce kymographs), and Plot *Z*-axis Profile (to measure fluorescence over time) functions in Fiji. Maximum difference projections were calculated as previously described (19), using the Duplicate, Stacks->Tools, Math->Subtract, and Z Project functions in Fiji. Maximum difference projection shows where fluorescence intensity increases most rapidly, which emphasizes exocytosis events and particle movement and deemphasizes static features that do not change during the course of imaging. Plots of average fluorescence intensity during exocytosis events were calculated using MATLAB (Mathworks). Image segmentation and 3D rendering of HSV-1 virus particle undergoing exocytosis from Rab6a vesicle were done in Imaris (Oxford Instruments) by constructing spots and surfaces for the objects at respective image slices. Access to the Imaris software was kindly provided by Drs. M. Foster Olive and Jessica Verpeut (Dept. of Psychology, Arizona State University).

### Statistics

Statistical comparisons of categorical data were performed using Fisher’s exact test in MATLAB (Mathworks). Standard error of the mean of categorical data was estimated by bootstrap resampling in MATLAB (Mathworks).

## Data Availability

All data and reagents are available upon request.

## SUPPLEMENTAL MATERIAL

Supplemental material jvi.00599-24-s0001.docx Supplemental figures, table, and methods.

Movie S1 jvi.00599-24-s0002.mp4 EmGFP-Rab6a and HSV-1 OK14 positive Vero cell showing mRFP-VP26 capsids in the nucleus and EmGFP-Rab6a in the Golgi region.

Movie S2 jvi.00599-24-s0003.avi EmGFP-Rab6a positive MRC5 cell. EmGFP-Rab6a is localized in the Golgi region and secretory vesicles accumulate in the cell periphery in the absence of active HSV-1 infection.

Movie S3 jvi.00599-24-s0004.mp4 EmGFP-Rab6a and HSV-1 OK14 positive Vero cell showing cotrafficking of Rab6a vesicles and HSV-1 capsids.

Movie S4 jvi.00599-24-s0005.avi An mCherry-Rab6a positive cell, an HSV-1 gM-pHluorin positive cell, and an mCherry-Rab6a and HSV-1 gM-pHluorin positive cell that all show accumulations of Rab6a vesicles or virus particles in the cell periphery.

Movie S5 jvi.00599-24-s0006.avi An mRFP-Rab6a and HSV-1 gM-pHluorin positive cell showing HSV-1 particle exocytosis from a tubular mCherry-Rab6a vesicle.

Movie S6 jvi.00599-24-s0007.avi HSV-1 exocytosis does not occur from mCherry-Rab5a vesicles and does not colocalized with mCherry-Rab5a in the cell periphery.

ASM does not own the copyrights to Supplemental Material that may be linked to, or accessed through, an article. The authors have granted ASM a non-exclusive, world-wide license to publish the Supplemental Material files. Please contact the corresponding author directly for reuse.
