# Supplementary material for: The Rab6 post-Golgi secretory pathway contributes to herpes simplex virus 1 (HSV-1) egress
Source: J Virol. 2024 Aug 13;98(9):e00599-24. doi: 10.1128/jvi.00599-24 (PMC11406995; doi:10.1128/jvi.00599-24)
Supplement: Supplemental material — Supplemental figures, table, and methods. [file jvi.00599-24-s0001.docx]

**The Rab6 Post-Golgi Secretory Pathway**

**Contributes to Herpes Simplex Virus 1 (HSV-1) Egress**

Melissa H. Bergeman, Kimberly Velarde, Hailee L. Hargis, Honor L. Glenn, Ian B. Hogue

**Supplemental Material 1**

Relative fluorescence over time of individual exocytosis events (n=45) of IH01 and mCherry-Rab6a in PK15 cells. Each exocytosis event is plotted (grey) with the exocytosis event aligned at t=0. Colored line represents the mean fluorescent intensity for gM-pHluorin (green) and mCherry-Rab6a (red).

Figure 1. HSV-1 Exocytosis Events from Rab6a Vesicles (n=28, 62%)


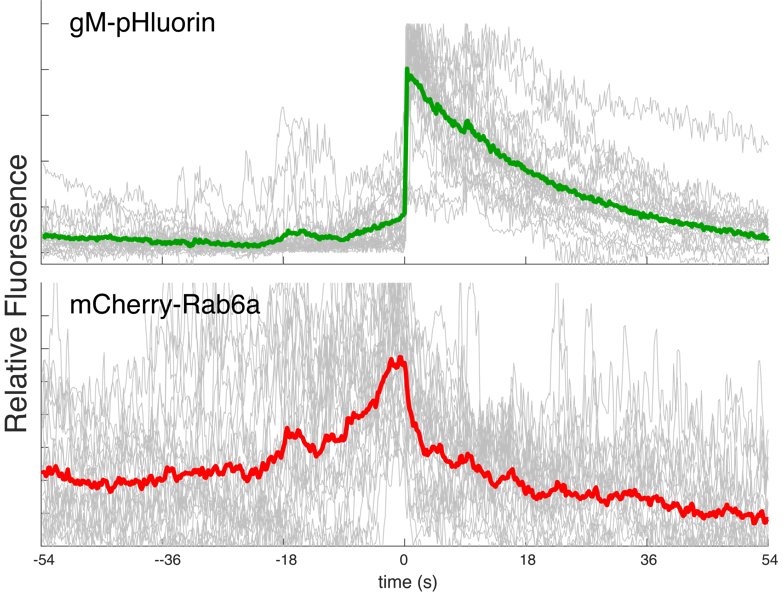


Figure 2. HSV-1 Exocytosis Events Not Associated with Rab6a (n=17, 37%)


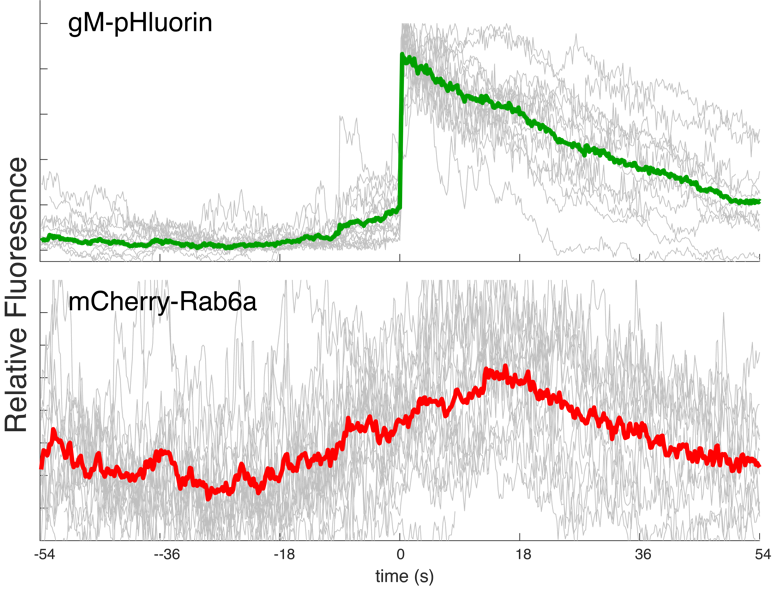


Figure 3. All HSV-1 Exocytosis Events (n=45)


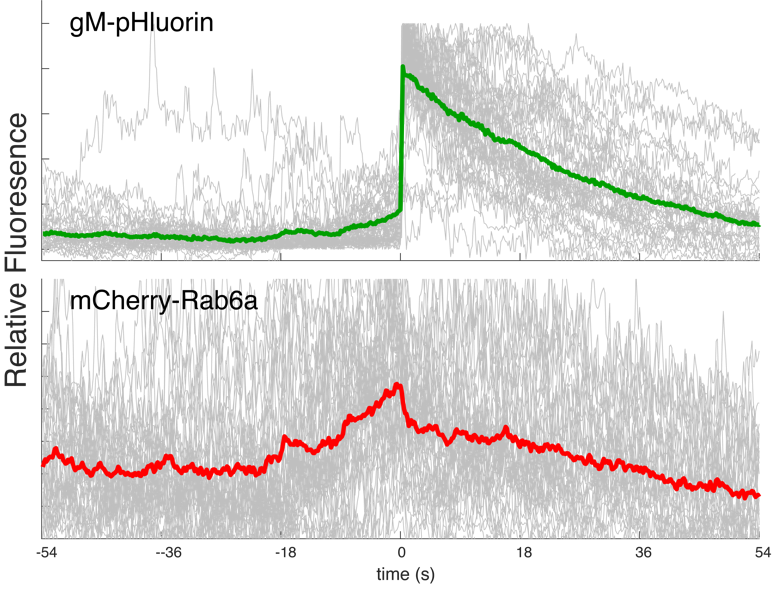


**Supplemental Material 2**

**Sequencing Rab6a(T27N)**

To ensure that the sequence of the Rab6a(T27N) construct is correct, adenovirus vector genomic DNA was isolated, the Rab6a(T27N) coding region was amplified by PCR, and Sanger sequencing was performed by the Arizona State University sequencing core facility. The sequencing chromatogram showing the desired mutation is shown (Supplemental Figure 1). The complete open reading frame was compared to the wild-type sequence using NCBI BLAST (Supplemental Figure 2). These data confirm that the Rab6a(T27N) transgene present in the adenovirus vector does contain the expected T27N mutation.


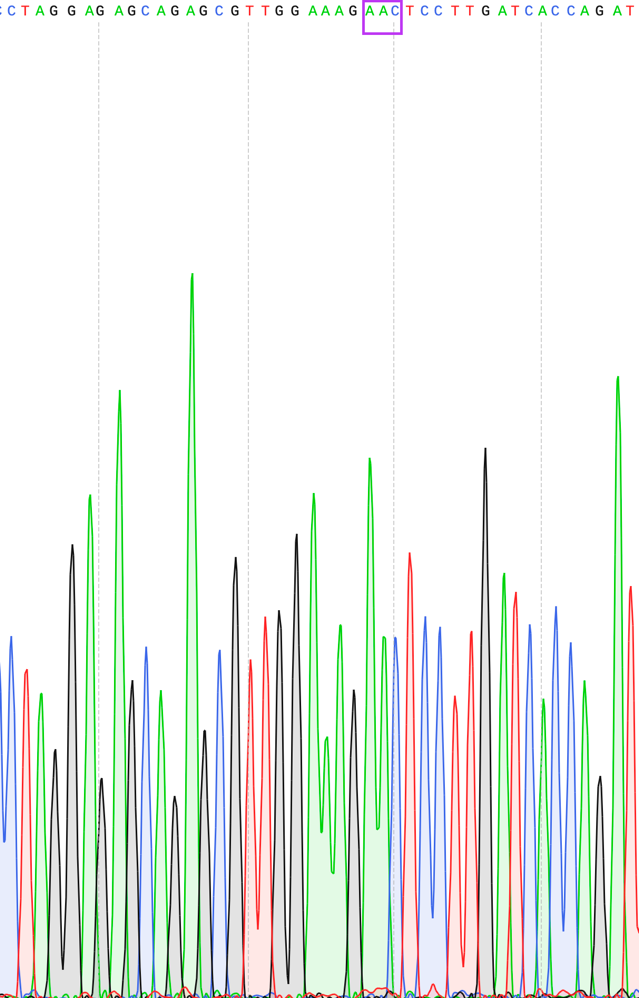


Supplemental Figure 1. Relevant portion of the Sanger sequencing chromatogram of the Rab6a(T27N) construct, showing the expected mutated codon (magenta box).

­


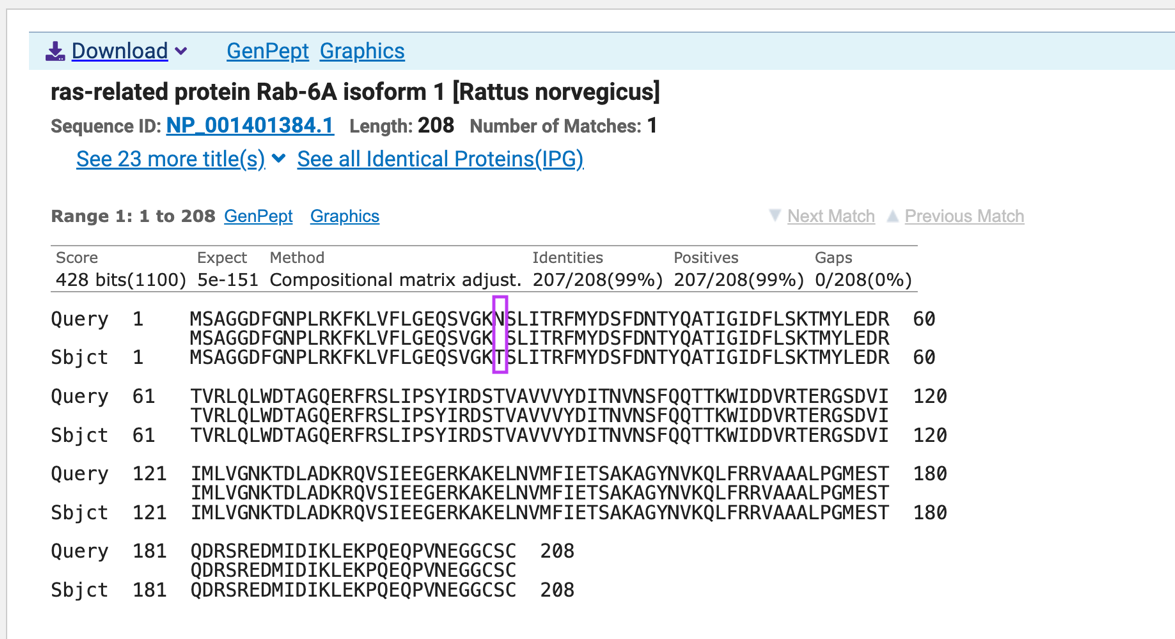


Supplemental Figure 2. NCBI BLAST protein sequence alignment of the sequenced Rab6a(T27N) construct compared to wild-type Rab6a. The T to N mutation is confirmed and highlighted (magenta box).

Materials and Methods

*DNA Extraction***.** Confluent HEK293A cells were infected with adenovirus vector Ad5-mCherry-Rab6a(T27N) and incubated until cytopathic effects and red fluorescence was detected across the entire monolayer. Supernatants were discarded, and the cells were scraped into 5mL of Phosphate Buffered Saline (PBS). The cells were pelleted by centrifuging at 2500 RPM for 2 minutes, and the supernatant was carefully discarded. 5 mL of cell lysis buffer (140 mM NaCl, 2 mM MgCl_2_, 0.5% Nonidet P-40, 200 mM Tris, pH 8.5) was added to the cell pellet and vortexed vigorously to resuspend. The lysate was then centrifuged at 2500 RPM for 2 minutes to pellet the nuclei. The supernatant was carefully discarded, and 2 mL of nuclear lysis solution (PBS, 1% SDS, pH 7.4) was added to the nuclei pellet, and incubated at 65°C for 10 minutes. 100 ug/mL of Proteinase K was added to the lysate, and incubated at 50°C for 60 minutes. The aqueous phase was extracted twice using phenol (pH 8.0) and once with 24:1 chloroform/isoamyl alcohol. Finally, DNA was precipitated using 20 uL of 1M sodium acetate and 4 mL of cold isopropanol, and then dissolved in Tris-EDTA (TE) buffer.

*PCR Reaction.* PCR primers (Supplemental Table 1) were designed, matching the mCherry and TK pA sequences flanking the Rab6a(T27N) coding region. PCR was performed in triplicate using Taq PCR Master Mix Kit (Qiagen), using 1.0 μg of extracted DNA. All PCR reactions were performed using the following conditions: 94°C for 3 min (initial denaturation); 35 cycles of 94°C for 1 minute, 59°C for 1 minute, 72°C for 2 minutes; followed by a final extension of 72°C for 10 minutes.

*Sequencing***.** PCR samples were purified using the QIAquick PCR purification kit (Qiagen). The purified PCR products were then sent for Sanger sequencing (ASU core facility) using the sequencing primer shown in Supplemental Table 1.

Supplemental Table 1: Primers

| **Primer Name** | **Primer Sequence** |
| --- | --- |
| Rab6T PCR F mCherry | 5’-GGACATCACCTCCCACAACG-3’ |
| Rab6T PCR R (TK pA) | 5’-GAACAAACGACCCAACACCCG-3’ |
| Rab6T Seq F mCherry | 5’-CGTGGAACAGTACGAACGCG-3’ |

**Supplemental Movies**

**Movie S1**. EmGFP-Rab6a and HSV-1 OK14 positive Vero cell showing mRFP-VP26 capsids in the nucleus and EmGFP-Rab6a in the Golgi region. Accumulations of EmGFP-Rab6a and mRFP-VP26 can be found at the cell periphery. Movie represents 36 seconds of imaging time and corresponds to Figure 2.

**Movie S2**. EmGFP-Rab6a positive MRC5 cell. EmGFP-Rab6a is localized in the Golgi region and secretory vesicles accumulate in the cell periphery in the absence of active HSV-1 infection. Move represents 1:40 min:sec imaging time and corresponds to Figure 3.

**Movie S3**. EmGFP-Rab6a and HSV-1 OK14 positive Vero cell showing cotrafficking of Rab6a vesicles and HSV-1 capsids. Movie represents 1:19 min:sec imaging time and corresponds to Figure 4.

**Movie S4**. An mCherry-Rab6a positive cell, an HSV-1 gM-pHluorin positive cell, and an mCherry-Rab6a and HSV-1 gM-pHluorin positive cell that all show accumulations of Rab6a vesicles or virus particles in the cell periphery. Movie represents 1:26 min:sec imaging time and corresponds to Figure 5.

**Movie S5**. An mRFP-Rab6a and HSV-1 gM-pHluorin positive cell showing HSV-1 particle exocytosis from a tubular mCherry-Rab6a vesicle. Movie represents 18 seconds imaging time and corresponds to Figure 6.

**Movie S6**. HSV-1 exocytosis does not occur from mCherry-Rab5a vesicles and does not colocalize with mCherry-Rab5a in the cell periphery. Movie represents 1:41 sec imaging time and corresponds to Figure 7.
